# Comparative Assessment of the Long-Term Effectiveness and Safety of Dapagliflozin and Empagliflozin as Add-on Therapy to Hypoglycemic Drugs in Patients with Type 2 Diabetes

**DOI:** 10.1155/2022/2420857

**Published:** 2022-05-23

**Authors:** Ai-Yu Yang, Hung-Chun Chen

**Affiliations:** ^1^Department of Pharmacy, Kaohsiung Medical University Hospital, Kaohsiung Medical University, Kaohsiung, Taiwan; ^2^Division of Nephrology, Department of Internal Medicine, Kaohsiung Medical University Hospital, Kaohsiung Medical University, Kaohsiung, Taiwan; ^3^Faculty of Medicine, College of Medicine, Kaohsiung Medical University, Kaohsiung, Taiwan

## Abstract

**Background:**

Sodium-glucose cotransporter 2 inhibitors (SGLT2i) reduce blood glucose, blood pressure, and body weight in patients with type 2 diabetes (T2D). However, the comparative long-term effectiveness and safety of SGLT2i among similar drugs, administered at different doses, have not been assessed. In this study, we compared the long-term effectiveness and safety of SGLT2i (dapagliflozin versus empagliflozin) as add-on therapy to hypoglycemic agents in T2D patients.

**Methods:**

This study was a single-center, 3-year, retrospective, observational study. For all patients in the study, drugs were evaluated for safety by documenting adverse drug reactions. The primary effectiveness was evaluated as the difference between hemoglobin A1c (HbA1c) values obtained at baseline and those obtained after 36 months of treatment. The proportion of participants with HbA1c levels <7.0% and <6.5% was also analyzed.

**Results:**

In total, 680 patients were enrolled in this study. Using propensity score matching, 234 patients each from the dapagliflozin and empagliflozin groups were selected based on patient characteristics. After 36 months of treatment, clinical parameters (including HbA1c, fasting plasma glucose, alanine aminotransferase, triglyceride levels, body weight, and systolic blood pressure) decreased significantly in these groups. The changes from the baseline for the physiological values and clinical parameters did not vary among the different dose groups of SGLT2i. The incidence of adverse drug reactions was approximately 7–8%. All patients with observed serious adverse reactions were hospitalized for urinary tract infections.

**Conclusion:**

Our study showed that the long-term continuous use of either dapagliflozin or empagliflozin as add-on therapy to hypoglycemic drugs for T2D patients is synergistically effective for lowering blood glucose, reducing body weight, and stabilizing blood pressure. Additionally, there was no significant difference in efficacy between dapagliflozin and empagliflozin, even with the administration of different doses of these agents.

## 1. Introduction

Sodium-glucose cotransporter 2 inhibitors (SGLT2i) form part of a new hypoglycemic drug class used to treat patients with type 2 diabetes (T2D). Large clinical trials have demonstrated that SGLT2i can decrease blood glucose, blood pressure, and body weight in T2D patients, with a prognostic benefit to the kidneys [1–3]. Previously, dapagliflozin (DPG) was limited to patients with an estimated glomerular filtration rate (eGFR) ≥60 mL/min/1.73 m^2^, whereas empagliflozin (EPG) could be used for those with an eGFR ≥45 mL/min/1.73 m^2^. Therefore, many patients were switched from DPG to EPG when the eGFR decreased. This switch was confirmed to be relatively safe by our previous study, which demonstrated that switching from DPG to EPG in T2D patients was still effective for maintaining blood glucose levels and caused no significant changes in renal function [4].

SGLT2i mitigate T2D by inhibiting renal glucose reabsorption and increasing glucose excretion in the urine [5]. However, increased urinary glucose excretion can promote the growth of pathogenic bacteria in the genitourinary tract, thereby increasing the risk of infection [6]. In randomized clinical trials of different doses of DPG (2.5/5/10 mg), urinary glucose concentrations increased with higher doses of the drug, but the incidence of urinary tract infections (UTIs) did not increase, and this rarely led to discontinuation of the drug [7]. A meta-analysis by Toyama et al. using a randomized trial showed an increased risk of genital mycotic infections (GMIs; RR = 2.86, 95% CI = 2.00–4.10) but not UTIs. The actual number of cases was relatively small, and the symptoms were easily controlled [8]. Furthermore, in a systematic evaluation by Caparrotta et al. using observational studies, SGLT2i exposure was found to be associated with GMIs (point estimate range hazard ratio [PER HR] = 2.08–3.15) and not with UTIs (PER HR = 0.72–0.98) [9]. However, the DARWIN (DApagliflozin Real World evIdeNce)-T2D multicenter retrospective study showed that the discontinuation of SGLT2i was usually due to genitourinary tract infections [10]. Both reproductive and urinary tract infections are widely known as side effects of SGLT2i, but their tendency to affect patients who discontinue the drug has shown conflicting results in randomized clinical trials versus real-world studies.

The eGFR declines at a rate of approximately 0.5–1 mL/min/1.73 m^2^ per year due to physiologic aging, with a more rapid decline of approximately 3 mL/min/1.73 m^2^ per year or more in T2D patients [1]. The DAPA-CKD, EMPA-REG OUTCOME, and CREDENCE trials all demonstrated that SGLT2i can slow the progression of renal disease [11–13]. In the DARWIN-T2D study, treatment with DPG for 6 months significantly reduced the albumin excretion rate (*P* = 0.045) compared with that in the control group (glucagon-like peptide 1 receptor agonist [GLP-1 agonist], dipeptidyl peptidase 4 inhibitor [DPP-4i] or gliclazide) but resulted in no difference in eGFR changes (*P* = 0.35) [14]. In the CVD-REAL 3 study, patients on SGLT2i had a slower rate of renal function decline compared to that in patients on other glucose-lowering drugs at a mean follow-up of 14.9 months [15]. In another Asian cross-national study comparing EPG and DPP-4is, with a mean follow-up of 5.7–6.8 months, the EPG group showed a significant reduction in all-cause mortality (HR = 0.64; 95% CI = 0.50–0.81) and end-stage renal disease (HR = 0.37; 95% CI = 0.24–0.58) [16]. However, to date, the observation period for most studies has been relatively short. In most countries, SGLT2i are limited to patients with an eGFR ≥60 mL/min/1.73 m^2^, limiting the availability of efficacy and safety data from patients at a high risk of renal function impairments [17]. Past studies have indicated that assessing changes in eGFR requires a longer observation period to explore trends [18].

T2D is a long-term, progressive disease that requires long-term treatment [18]. In the real world, hypoglycemic drugs or doses are often changed owing to efficacy or side effects. SGLT2i have become commonly used drugs; however, the comparative efficacy and safety of SGLT2i among similar drugs at different doses are not known. In this study, we compared the long-term effectiveness and safety of SGLT2i (DPG versus EPG) as an add-on to hypoglycemic drugs in T2D patients.

## 2. Methods

### 2.1. Patients

This was a retrospective cohort study comprising adult patients (aged ≥20 years) who had been receiving treatment at the Kaohsiung Medical University Hospital (KMUH). KMUH is a medical center located in southern Taiwan with approximately 1600 beds and 6000 patient visits per day. This study was approved by the Institutional Review Board of Kaohsiung Medical University Hospital (KMUHIRB-E(I)-20190349). The study population included outpatients with T2D who started using DPG or EPG between January 1, 2015, and June 30, 2018. This study was based on previously conducted studies and does not describe any experiments involving human participants or animals conducted by any of the authors.

**Figure 1:**
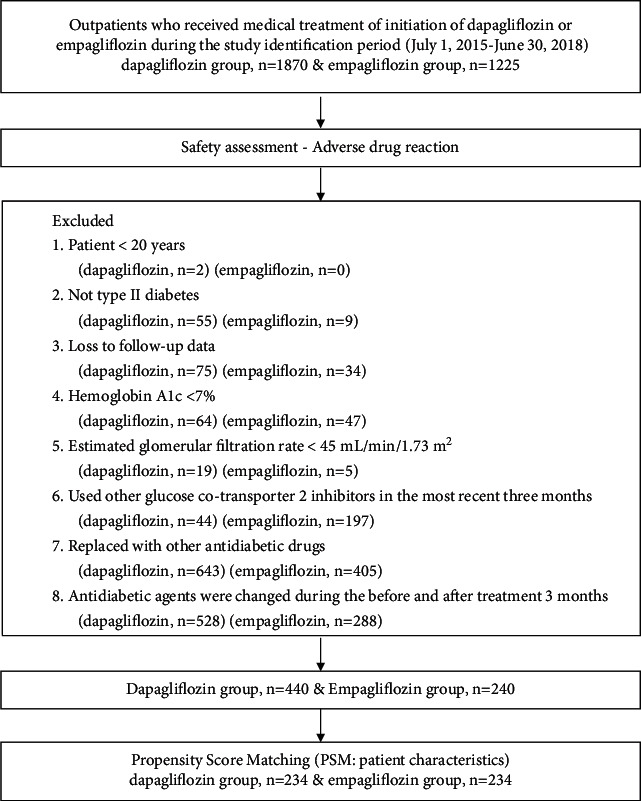
Flow chat of patient selection.

The inclusion criteria were as follows: (1) patients aged ≥20 years with eGFR ≥45 mL/min/1.73 m^2^, (2) T2D, and (3) hemoglobin A1c (HbA1c) ≥7%. The exclusion criteria were as follows: (1) missing HbA1c or eGFR data before or after treatment, (2) acute kidney failure (drastic changes in renal function) within 2 weeks before treatment, (3) kidney transplant, (4) pregnancy or lactation, (5) concurrent anticancer or immunosuppressive treatment, and (6) the use of other SGLT2i in the most recent 3 months. A flow diagram of patient selection is shown in Figure 1. DPG treatment (dapagliflozin 10 mg/tab) was introduced at KMUH in September 2015, whereas EPG treatment (empagliflozin 25 mg/tab) was introduced in October 2016. The last date of data capture was December 2020, and patients were enrolled for a maximum follow-up of 36 months.

### 2.2. Procedure

Patient data were collected using the electronic medical record system of the hospital, which included demographic data (age and sex), medical history (mainly focused on cardiovascular diseases), and the use of other hypoglycemic drugs. For all patients in the study, drugs were evaluated for safety. Analysis was performed by reviewing the electronic medical record for adverse drug reaction notes and the prescriber's clinical notes comprising reasons for discontinuation.

We defined the efficacy control target as HbA1c<7%. The primary effectiveness was evaluated as the difference between HbA1c values obtained at baseline and those obtained after 36 months of treatment. Secondary outcomes included changes in body weight (BW), systolic and diastolic blood pressure (SBP and DBP, respectively), fasting plasma glucose (FPG), alanine aminotransferase (ALT), renal function (expressed as eGFR), and lipid profiles (total cholesterol, Chol (T); triglyceride, TG; low-density lipoprotein-cholesterol level, LDL-C; high-density lipoprotein-cholesterol level, HDL-C). Occurrences of new coronary artery disease (CAD), heart failure (HF), stroke, and hospitalization for heart failure (hHF) were also monitored.

The dosage of SGLT2i can also be a factor that affects efficacy; therefore, the clinical response of patients using different doses was also analyzed. Since the study only utilized dapagliflozin 10 mg/tab and empagliflozin 25 mg/tab, the dose grouping was based on “one pill” and “half a pill.” In addition, patients' prescriptions are often adjusted based on the posttreatment responses. The initial dose can be changed, and the final grouping was based on the dose used at the time of post-treatment data collection and the dose that had been maintained for more than 3 months.

### 2.3. Statistical Analyses

A propensity score model (PSM) was used to match patients receiving DPG to those receiving EPG based on patient characteristics. The Kolmogorov–Smirnov test was used initially to assess whether the quantitative data were normally distributed. Here, the quantitative variables are expressed as the median and interquartile range, 25–75%, and qualitative variables are expressed as numbers and percentages. The chi-square test was used to compare qualitative data (including sex and cardiovascular history), and Fisher's test was used for any value less than 5.

The nonparametric Wilcoxon sign rank test was used to compare the differences in quantitative variables before and after treatment with SGLT2i. The Mann–Whitney *U* test was used to compare the quantitative variables between the high- and low-dose groups of DPG and EPG. A least squares model was used to evaluate the association between the demographic and baseline parameters of the patients and the changes in the HbA1c levels (*Δ*HbA1c) at 6 months after the of initiation therapy. Multiple regression analyses were performed to analyze the factors that showed a significant association with each dependent variable. *P* values <0.05 were considered statistically significant.

## 3. Results

### 3.1. Patient Characteristics

In total, 3095 patients initiated SGLT2i treatment. Of these patients, 1870 were treated with DPG and 1225 with EPG. Ultimately, 680 patients were enrolled in this study. Patients who initiated DPG were relatively younger and had better kidney function than those who initiated EPG. After PSM, 234 patients each from the DPG and EPG groups were selected (a flow diagram is presented in Figure 1).

In both the DPG and EPG groups, there were more males than females. Hypertension and hyperlipidemia were the most common cardiovascular comorbidities observed in both groups. The types of other hypoglycemic drugs concurrently used were also important factors affecting the efficacy. The baseline values of other clinical parameters are shown in Table 1.

### 3.2. Effectiveness

We investigated 122 treated patients for 36 months. After 36 months of treatment, clinical parameters (including FPG, HbA1c, ALT, TG, BW, and SBP) decreased significantly in the DPG and EPG groups. A comparison of the parameters in the groups between those measured at baseline and after 36 months revealed no significant differences (Table 2). In the real-world study, the proportion of missed follow-ups was too high as the follow-up period became longer, and the bias caused by the small amount of data could be analyzed. This study additionally compared the data obtained after 6 months of treatment (Supplementary Table 1). The results showed no statistical difference between the baseline and 6-month values in the different groups.

**Figure 2:**
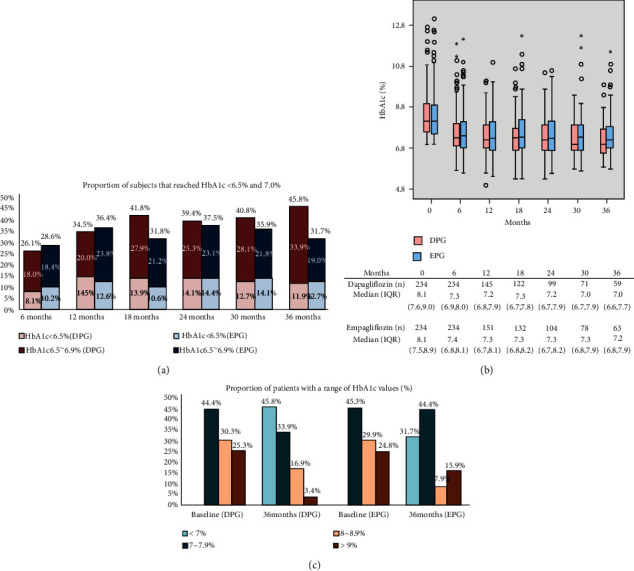
Long-term tracking of HbA1c-related data; HbA1c: hemoglobin A1c; DPG: dapagliflozin; EPG: empagliflozin. (a) Proportion of subjects that reached HbA1c< 6.5% (DPG), HbA1c 6.5~6.9% (DPG), HbA1c< 6.5% (EPG), and HbA1c 6.5~6.9% (EPG) at 6, 12, 18, 24, 30, and 36 months. (b) Box plots indicating the HbA1c values compared to baseline levels after 6, 12, 18, 24, 30, and 36 months of treatment. (c) Proportion of subjects with a range of HbA1c values at 0 and 36 months. Range of HbA1c values by bars: < 7.0%, 7%~7.9%, 8%~8.9%, > 9%.

After using SGLT2i (DPG and EPG) as an additional treatment with other hypoglycemic drugs, the percentage of patients who achieved treatment goals <7% is recorded in Figure 2(a). Although there was a slight difference between the two treatment groups with respect to the proportion who achieved their treatment goal, there was a significant difference in the decline from baseline values at months 6, 12, 18, 24, 30, and 36 (Figure 2(b)). Nearly half of the baseline HbA1c values were 7–7.9% in both the DPG and EPG groups before treatment, and the proportion with HbA1c<7% was significantly higher in the DPG group than in the EPG group after 36 months of treatment (Figure 2(c)). The *Δ*HbA1c showed a significant negative correlation with BW and FPG, HbA1c, and eGFR levels at baseline. Multiple regression analyses showed that the baseline HbA1c value might be the main influencing factor (Table 3).

### 3.3. Dosage of SGLT2i

The dosage of SGLT2i possibly influences the efficacy of therapy. The changes from baseline values for the demographics (BW, SBP, and DBP) and clinical parameters (FPG, HbA1c, ALT, Scr, eGFR, Chol(T), TG, LDL-C, HDL-C) were not different for the different dose groups of SGLT2i. No significant differences between the parameters of the groups measured at baseline and those measured at 36 months were observed (Table 4). After 36 months of follow-up, it was possible to analyze the bias caused by the small amount of data and compare the data for 6 months of treatment (Supplementary Table 2). The results showed that there was no difference in blood glucose, blood pressure, and BW between the administration of one tablet and half a tablet.

### 3.4. Cardiovascular Events and Mortality

Cardiovascular events included new onset of CAD (*N* = 15, 3.2%, DPG vs. EPG = 7 vs. 8), HF (*N* = 4, 0.8%, 2 vs. 2), stroke (*N* = 5, 1.1%, 2 vs. 3), and hHF (*N* = 2, 0.4%, 2 vs. 0), which all occurred during the observation period.

### 3.5. Safety

All patients in the study were evaluated for safety by documenting adverse drug reactions. Adverse drug reactions were documented in 134 (7.2%) patients using DPG and 95 (7.7%) patients using EPG during the study period (Table 5). The majority of patients experienced symptoms during the first 3 months of treatment, with only 18 patients in the DPG group and 10 patients in the EPG group experiencing adverse reactions after 3 months.

**Figure 3:**
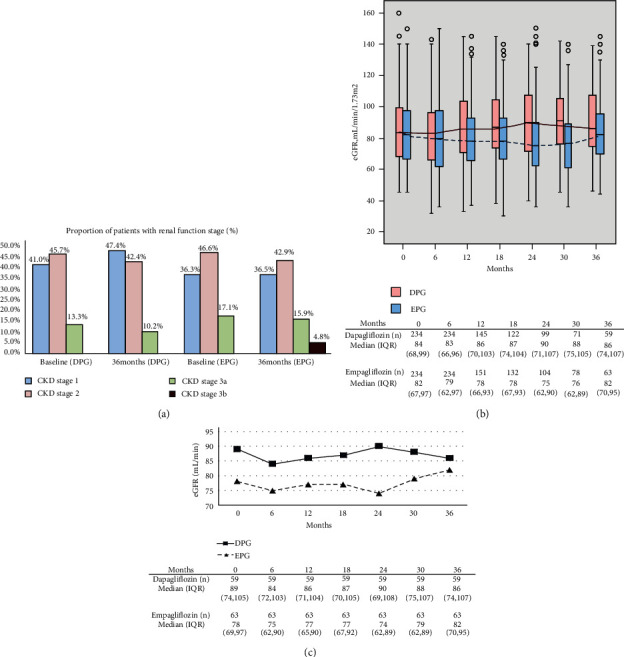
Long-term tracking of eGFR-related data; eGFR: estimated glomerular filtration rate (mL/min/1.73 m^2^); DPG: dapagliflozin; EPG: empagliflozin. (a) Proportion of patients with different renal function stages at 0 and 36 months. Range of eGFR values by bars: CKD stage 1: ≥ 90; CKD stage 2: 60~89; CKD stage 3a: 45~59; CKD stage 3b: 30~44 (mL/min/1.73 m^2^). (b) Box plots indicate the eGFR values compared to baseline levels after 6, 12, 18, 24, 30, and 36 months of treatment. (c) Change in clinical parameters from baseline values to 36 months for long-term users.

In terms of the serious adverse reactions, three patients in the DPG group and one patient in the EPG group were hospitalized due to a severe UTI. While analyzing the characteristics of patients with adverse events (*N* = 229), it was observed that their median age was significantly higher (63 vs. 60, *P* = 0.034) than that of event-free patients (*N* = 2,866). The proportion of females was also significantly higher (72.1% vs. 42.2%). After 36 months of treatment, the proportion of patients with CKD stage 3 (30–59 mL/min/1.73 m^2^) was higher than that before treatment only in the EPG group (Figure 3(a)). Long-term follow-up revealed that renal function showed a downward trend in the EPG group (*y* = −0.3214*x* + 79.857), as opposed to an upward trend in the DPG group (*y* = 0.7143*x* + 83.429) (Figure 3(b)).

## 4. Discussion

### 4.1. Effectiveness

Our study showed that the long-term use of SGLT2i (DPG and EPG) as add-on therapy for T2D patients significantly reduced blood glucose, BW, and blood pressure. Additionally, there was no significant difference in efficacy between the two, even with the administration of different doses of these agents. These results were consistent with those of the network meta-analysis conducted by Shyangdan et al. [19]. In addition, a comparative study in Italy also showed that dose differences did not affect the hypoglycemic effect, which leads to the assumption that ethnic differences (neither Caucasian populations nor in Asians) do not affect the hypoglycemic effect [20].

A study by Reifsnider et al. demonstrated that EPG was a more cost-effective treatment than DPG and canagliflozin in patients with T2D and established cardiovascular disease (CVD) [21]. Dutta et al. compared EPG 10 mg, 12.5 mg (half EPG 25), and 25 mg for their efficacy in lowering blood glucose (−0.9, −1.0, −1.0; *P* = 0.363) and suggested that half a tablet of EPG-25 is the most cost-effective method when using EPG [22]. Our results showed no difference in the effects of the SGLT2i analogs (DPG or EPG) and their dosages (one pill or half a pill) on blood glucose, blood pressure, and BW; however, our findings did not provide sufficient evidence for long-term cardiovascular benefits, and therefore, more evidence from a large randomized controlled trial is needed.

### 4.2. Cardiovascular Outcome

The DECLARE-TIMI 58, EMPA-REG OUTCOME, and CANVAS trials provide evidence of the cardiovascular-protective effect of SGLT2i [1–3]. All patients in the EMPA-REG OUTCOME trial had established CVD, and the DECLARE-TIMI 58 trial involved patients with cardiovascular risk factors [1, 2]. Both studies showed a reduction in hHF. However, the DECLARE-TIMI 58 study showed that DPG was effective in reducing hHF in patients without a history of atherosclerotic cardiovascular diseases (ASCVD) or HF [1, 2].

A real-world retrospective study found that for patients without ASCVD, DPG was more effective in reducing hHF than EPG (HR: 0.67, 95% CI: 0.49–0.90). There was no such difference in the group with ASCVD (HR: 1.12, 95% 0.87–1.45) [23]. Owing to the limited number of cases in our study, it is difficult to assess whether differences between the similar drugs (DPG or EPG) might affect the incidence of cardiovascular events. Based on randomized trials and real-world evidence, it is important to consider a history of ASCVD in the clinical use of DPG and EPG.

### 4.3. Renal Outcome

The CVD-REAL 3 study showed a slower decline in renal function with SGLT2i compared to that with other hypoglycemic drugs [15]. In our long-term follow-up, only the EPG group showed a decreasing trend in renal function, whereas the DPG group showed an increasing trend. This could be related to the publication of SGLT2i-related renal prognostic studies during the study follow-up period, which influenced their clinical use.

If the clinical data parameters of the long-term users were exclusively analyzed (Figure 3(c)), both the DPG (*y* = 0.1071*x* + 86.714) and EPG (*y* = 0.6071*x* + 75) groups showed an increasing trend. The discrepancy in the data could be related to the fact that the baseline eGFR values for patients subjected to long-term use differed from those of all patients (higher in the DPG group and lower in the EPG group). Current randomized clinical trial studies have shown that SGLT2i reduce renal adverse events regardless of baseline eGFR and albuminuria values [24]. In patients with T2D, SGLT2i are undoubtedly of great benefit for the prognosis of renal function.

### 4.4. Safety

In our study, the incidence of UTIs and GMIs was higher, and the proportion of women affected was significantly higher. Amos et al. showed that canagliflozin exposure was associated with the occurrence of GMIs, but only females had a higher incidence when sex was analyzed [25]. The pooled studies from randomized controlled trials also show that the use of DPG causes UTIs more commonly in the elderly and in women [7]. It is thought that this might be related to the physiology of women, who are more prone to urinary tract and reproductive organ infections due to the use of SGLT2i. The serious adverse reactions in the DPG and EPG groups were UTIs leading to hospitalization, suggesting that increased health education prior to SGLT2i use could be needed to reduce the severity of adverse reactions. At the last follow-up date, only five patients had expired. One patient died of CVD, and the other four died of cancer.

### 4.5. Limitations

There are several limitations owing to the retrospective design of this study. The first is the lack of documentation of patients' diets and lifestyle habits. Second, although the data were analyzed after matching according to patient characteristics, it is not possible to exclude possible bias owing to exclusion conditions or matching. Third, patients' tolerance to side effects varies greatly, and minor adverse reactions might have been overlooked or omitted from the records. In addition, this study was limited to a medical center with a limited number of cases, and it is difficult to track values regularly in a real-world study. The longer the follow-up period, the more observations are missed, and the limited data can cause statistical bias. This study was conducted with limited data to provide clinical real-world data for analysis.

## 5. Conclusion

Our study demonstrated that the use of either DPG or EPG as add-on therapy to hypoglycemic drugs in patients with T2D was effective in synergistically lowering blood glucose. The treatment option also showed benefits for weight reduction and blood pressure stabilization. In addition, there was no significant difference in efficacy between DPG and EPG. There was also no significant difference in the efficacy of different doses. GMIs and UTIs were the most common adverse reactions observed during clinical use, and there should be an emphasis on lifestyle education before initiation of treatment to reduce the incidence and severity of adverse events.

## Figures and Tables

**Table 1:** Baseline demographics and clinical parameters of study participants.

	Dapagliflozin (*n* = 234)	Empagliflozin (*n* = 234)	*P* value
Male, n (%)	122 (52.1%)	136 (58.1%)	0.193
Age, years	63 (55, 67)	61 (54, 68)	0.337
Body weight, kg	72.4 (63.5, 81.4)	70.9 (62.4, 82.2)	0.592
SBP, mmHg	141 (127, 152)	140 (128, 151)	0.986
DBP, mmHg	78 (72, 87)	80 (74, 88)	0.082
FPG, mg/dL	165 (143, 187)	161 (136, 189)	0.734
HbA1c, %	8.1 (7.6, 9.0)	8.1 (7.5, 8.9)	0.536
ALT, IU/L	27 (21, 39)	26 (20, 39)	0.943
Scr, mg/dL	0.84 (0.65, 1.01)	0.87 (0.69, 1.03)	0.136
eGFR, mL/min/1.73 m^2^	83.6 (68.3, 99.2)	82.0 (67.2, 97.1)	0.321
CHOL(T), mg/dL	158 (137, 179)	168 (144, 189)	0.003∗
Triglyceride, mg/dL	126 (89, 180)	127 (89, 178)	0.903
LDL-C, mg/dL	86 (71, 103)	93 (76, 113)	0.001∗
HDL-C, mg/dL	43 (36, 50)	44 (36, 54)	0.294
*Co morbidities, n(%)*			
Hypertension	161 (68.8%)	142 (60.7%)	0.066
Dyslipidemia	189 (80.8%)	168 (71.8%)	0.022∗
History of stroke/TIA	16 (6.8%)	26 (11.1%)	0.106
CAD (MI, IHD)	37 (15.8%)	35 (15.0%)	0.798
Heart failure	10 (4.3%)	13 (5.6%)	0.521
Heart arrhythmia	8 (3.4%)	10 (4.3%)	0.631
*Concomitant medication, n(%)*			
Metformin	132 (56.4%)	145 (62.0%)	0.221
Sulfonylurea	137 (58.5%)	129 (55.1%)	0.455
Pioglitazone	62 (26.5%)	68 (29.1%)	0.536
DPP-4 inhibitor	130 (55.6%)	83 (35.5%)	<0.001∗
Acarbose	30 (12.8%)	20 (8.5%)	0.135
Insulin	74 (31.6%)	68 (29.1%)	0.546
GLP-1 agonist	6 (2.6%)	11 (4.7%)	0.217

Data are shown as median (IQR). ALT: alanine aminotransferase; CAD: coronary artery disease; CHOT(T): total cholesterol; DBP: diastolic blood pressure; DDP-4: dipeptidyl peptidase-4; eGFR: estimated glomerular filtration rate; FPG: fasting plasma glucose; GLP-1: glucagon-like peptide-1; HbA1c: hemoglobin A1c; HDL-C: high-density lipoprotein- cholesterol level; IHD: ischemic heart disease; LDL-C: low-density lipoprotein-cholesterol level; MI: myocardial infarction; SBP: systolic blood pressure; Scr: serum creatinine; TIA: transient ischemic attack. ∗*p* value <0.05 were considered statistically significant.

**Table 2:** Changes in clinical parameters from baseline with dapagliflozin or empagliflozin.

	Dapagliflozin (*n* = 59)			Empagliflozin (*n* = 63)			*P* value‡
	Baseline	36 months	*P* value†	Baseline	36 months	*P* value†	
HbA1c, %	8.0 (7.6, 8.6)	7.0 (6.6, 7.7)	<0.001∗	7.9 (7.4, 9.3)	7.2 (6.8, 7.9)	<0.001∗	0.324
Body weight, kg	75 (66, 84)	73 (66, 80)	<0.001∗	74 (64, 84)	71 (62, 80)	<0.001∗	0.798
SBP, mmHg	142 (131, 155)	131 (120, 140)	<0.001∗	144 (129,150)	132 (120, 140)	<0.001∗	0.379
DBP, mmHg	80 (74, 89)	80 (72, 87)	0.228	81 (73, 87)	77 (68, 82)	<0.001∗	0.081
FPG, mg/dL	161 (137, 186)	136 (117, 156)	<0.001∗	167 (134, 188)	126 (113, 156)	<0.001∗	0.171
ALT, IU/L	30 (23, 43)	24 (19, 37)	<0.001∗	26 (20, 36)	23 (19, 30)	0.005∗	0.158
Scr, mg/dL	0.79 (0.60, 0.95)	0.79 (0.61, 0.99)	0.228	0.90 (0.74, 1.02)	0.90 (0.75, 1.07)	0.174	0.998
eGFR, mL/min/1.73 m^2^	89 (74, 105)	86 (74, 107)	0.898	78 (69, 97)	82 (70, 95)	0.723	0.933
CHOL(T), mg/dL	150 (128, 173)	152 (136, 165)	0.913	161 (138, 183)	153 (131, 179)	0.064	0.150
Triglyceride, mg/dL	127 (97, 177)	110 (76, 162)	0.031∗	115 (96, 174)	107 (74, 160)	0.037∗	0.907
LDL-C, mg/dL	82 (66, 96)	80 (71, 98)	0.846	90 (72, 111)	83 (62, 97)	0.019∗	0.059
HDL-C, mg/dL	43 (37, 49)	42 (37, 53)	0.275	40 (34, 50)	43 (37, 52)	0.194	0.718

Data are shown as median (IQR). ALT: alanine aminotransferase; CHOT(T): total cholesterol; DBP: diastolic blood pressure; eGFR: estimated glomerular filtration rate; FPG: fasting plasma glucose; HbA1c: hemoglobin A1c; HDL-C: high-density lipoprotein-cholesterol level; LDL-C: low density lipoprotein-cholesterol level; SBP: systolic blood pressure; Scr: serum creatinine. †Wilcoxon sign rank test was used to compare of differences within groups measured at baseline and 36 months. ‡Mann–Whitney *U* test was used to compare of differences between groups measured at baseline and 36 months. ∗*p* value <0.05 were considered statistically significant.

**Table 3:** Relationship between the *Δ*HbA1c and the clinical parameters at baseline.

	*Δ*HbA1c (univariate)		*Δ*HbA1c (multivariate)	
	*r*	*P*	*β*	*P*
Gender, female	0.063	0.177		
Age, years	0.117	0.011∗	0.903	0.367
Body weight	-0.101	0.030∗	-1.495	0.136
Systolic blood pressure	0.053	0.252		
Diastolic blood pressure	0.036	0.440		
Baseline FPG	-0.307	<0.001∗	-0.111	0.912
Baseline HbA1c	-0.482	<0.001∗	-12.46	<0.001∗
Baseline eGFR	-0.092	0.047∗	-0.875	0.382
Hypertension	0.042	0.363		
Dyslipidemia	0.004	0.937		
History of stroke/TIA	-0.059	0.201		
CAD (MI, IHD)	0.082	0.075		
Heart failure	-0.004	0.927		
Heart arrhythmia	-0.019	0.686		
Metformin	0.104	0.024∗	0.840	0.401
Sulfonylurea	-0.001	0.981		
Pioglitazone	0.003	0.944		
DDP-4 inhibitor	0.010	0.830		
Acarbose	0.017	0.722		
Insulin	-0.035	0.447		
GLP-1 agonist	0.089	0.053		

CAD: coronary artery disease; DDP-4: dipeptidyl peptidase-4; eGFR: estimated glomerular filtration rate; FPG: fasting plasma glucose; GLP-1: glucagon-like peptide-1; HbA1c: hemoglobin A1c; IHD: ischemic heart disease; MI: myocardial infarction; TIA: transient ischemic attack. ∗*p* value <0.05 were considered statistically significant (∗ upper index).

**Table 4:** Comparison changes in clinical parameters from baseline between different doses.

	One pill (*n* = 91)			Half a pill (*n* = 31)			*P* value‡
	Baseline	36 months	*P* value†	Baseline	36 months	*P* value†	
HbA1c, %	8.0 (7.4, 8.9)	7.0 (6.7, 7.9)	<0.001∗	7.8 (7.5, 9.4)	7.3 (6.9, 7.7)	<0.001∗	0.700
Body weight, kg	75 (64, 86)	73 (63, 82)	<0.001∗	74 (66, 78)	70 (65, 76)	0.001∗	0.098
SBP, mmHg	142 (131, 152)	132 (121, 139)	<0.001∗	144 (133,152)	131 (119, 141)	0.001∗	0.881
DBP, mmHg	82 (74, 89)	78 (70, 86)	0.007∗	77 (73, 88)	77 (67, 82)	0.029∗	0.946
FPG, mg/dL	165 (142, 184)	130 (116, 158)	<0.001∗	160 (131, 190)	128 (113, 155)	0.001∗	0.712
ALT, IU/L	29 (22, 41)	23 (19, 30)	<0.001∗	26 (19, 37)	23 (19, 39)	0.008∗	0.902
Scr, mg/dL	0.87 (0.66, 1.02)	0.88 (0.67, 1.06)	0.160	0.85 (0.65, 0.93)	0.80 (0.71, 0.97)	0.264	0.858
eGFR, mL/min/1.73 m^2^	83 (69, 98)	84 (68, 104)	0.639	87 (74, 103)	82 (76, 97)	0.868	0.687
CHOL(T), mg/dL	153 (135, 178)	151 (129, 172)	0.436	165 (134, 177)	157 (136, 170)	0.247	0.631
Triglyceride, mg/dL	128 (97, 190)	112 (77, 177)	0.016∗	113 (96, 149)	99 (70, 137)	0.061	0.924
LDL-C, mg/dL	83 (69, 104)	82 (62, 97)	0.370	92 (72, 105)	83 (72, 102)	0.108	0.357
HDL-C, mg/dL	40 (34, 49)	42 (36, 51)	0.128	43 (36, 50)	47 (40, 54)	0.405	0.981

Data are shown as median (IQR). ALT: alanine aminotransferase; CHOT(T): total cholesterol; DBP: diastolic blood pressure; eGFR: estimated glomerular filtration rate; FPG: fasting plasma glucose; HbA1c: hemoglobin A1c; HDL-C: high-density lipoprotein-cholesterol level; LDL-C: low-density lipoprotein-cholesterol level; SBP: systolic blood pressure; Scr: serum creatinine. †Wilcoxon sign rank test was used to compare of differences within groups measured at baseline and 36 months. ‡Mann–Whitney *U* test was used to compare of differences between groups measured at baseline and 36 months. ∗*p* value <0.05 were considered statistically significant.

**Table 5:** Adverse events.

	Dapagliflozin	Empagliflozin
Total adverse events	134	95
Serious adverse events	3	1
Patient had more than one adverse event	9	8
AEs leading to discontinuation	116 (86.6%)	85 (89.5%)
Genital mycotic infections	19 (14.2%)	23 (24.2%)
Urinary tract infections	26 (19.4%)	17 (17.9%)
Hypotension	1 (0.8%)	2 (2.1%)
Dizziness	10 (7.5%)	9 (9.5%)
Skin itching/eruption	13 (9.7%)	8 (8.4%)
Hypoglycemia	0	0
Diabetic ketoacidosis	3 (2.2%)	0
Renal impairment	1 (0.8%)	3 (3.2%)
Gastrointestinal symptoms†	16 (11.9%)	8 (8.4%)
Lower urinary tract symptoms††	18 (13.4%)	22 (23.1%)
Others‡	33 (24.6%)	11 (11.6%)

†Gastrointestinal symptoms: abdominal fullness, abdominal pain, constipation, diarrhea, gastroesophageal reflux disease, nausea, and vomiting. ††Lower urinary tract symptoms: dysuria, frequent urination, nocturia, polyuria, and urination discomfort. ‡Others: acute pyelonephritis, discomfort, edema, foam urine, headache, hematuria, hunger, lumbar pain, muscle pain, palpitation, chest tightness, weakness, and weight loss.

## Data Availability

Data sharing is not applicable to this article as no new data were created or analyzed in this study.

## Supplementary Materials

Supplementary Materials Supplementary Table 1: changes in clinical parameters from baseline with dapagliflozin or empagliflozin. Supplementary Table 1: changes in clinical parameters from baseline with dapagliflozin or empagliflozin.
