# Supplementary material for: Comparative Assessment of the Long-Term Effectiveness and Safety of Dapagliflozin and Empagliflozin as Add-on Therapy to Hypoglycemic Drugs in Patients with Type 2 Diabetes
Source: J Diabetes Res. 2022 May 23;2022:2420857. doi: 10.1155/2022/2420857 (PMC9152409; doi:10.1155/2022/2420857)
Supplement: Supplementary Materials — Supplementary Table 1: changes in clinical parameters from baseline with dapagliflozin or empagliflozin. Supplementary Table 1: changes in clinical parameters from baseline with dapagliflozin or empagliflozin. [file 2420857.f1.docx]

**Supplementary table 1 - Changes in clinical parameters from baseline with dapagliflozin or empagliflozin**

|  | **Dapagliflozin (n=234)** | |  |  | **Empagliflozin (n=234)** | |  | **P-value**‡ |
| --- | --- | --- | --- | --- | --- | --- | --- | --- |
|  | **Baseline** | **6 months** | **P-value**† |  | **Baseline** | **6 months** | **P-value**† |  |
| HbA1c, % | 8.1 (7.6, 9.0) | 7.3 (6.9, 8.0) | <0.001* |  | 8.1 (7.5, 8.9) | 7.4 (6.8, 8.1) | <0.001* | 0.653 |
| Body weight, kg | 72 (63, 81) | 70 (61, 81) | <0.001* |  | 71 (62, 82) | 70 (62, 82) | <0.001* | 0.766 |
| SBP, mmHg | 141 (127, 152) | 136 (125, 147) | 0.005* |  | 140 (128,151) | 133 (124, 148) | <0.001* | 0.455 |
| DBP, mmHg | 78 (72, 87) | 78 (71, 87) | 0.394 |  | 80 (74, 88) | 78 (71, 86) | 0.001* | 0.083 |
| FPG, mg/dL | 165 (143, 187) | 140 (121, 160) | <0.001* |  | 161 (136, 189) | 134 (112, 153) | <0.001* | 0.436 |
| ALT, IU/L | 27 (21, 39) | 23 (18, 27) | <0.001* |  | 26 (20, 39) | 21 (17, 26) | 0.005 | 0.646 |
| eGFR, mL/ min/ 1.73 m^2^ | 84 (68, 99) | 83 (66, 96) | 0.001* |  | 82 (67, 97) | 79 (62, 97) | 0.074 | 0.367 |
| CHOL(T), mg/dL | 158 (137, 179) | 154 (135, 179) | 0.826 |  | 168 (144, 190) | 162 (142, 186) | 0.004* | 0.066 |
| Triglyceride, mg/ dL | 126 (89, 180) | 112 (74, 160) | <0.001* |  | 127 (89, 178) | 114 (78, 161) | 0.002* | 0.554 |
| LDL-C, mg/dL | 86 (71, 103) | 83 (69, 101) | 0.415 |  | 93 (76, 113) | 90 (75, 105) | 0.004* | 0.117 |
| HDL-C, mg/dL | 43 (36, 50) | 43 (36, 50) | 0.642 |  | 44 (36, 54) | 46 (37, 53) | 0.540 | 0.986 |

Data are shown as Median (IQR). ALT, alanine aminotransferase; CHOT(T), total cholesterol; DBP, diastolic blood pressure; eGFR, estimated glomerular filtration rate; FPG, fasting plasma glucose; HbA1c, Hemoglobin A1c; HDL-C, high-density lipoprotein- cholesterol level; LDL-C, low density lipoprotein- cholesterol level; SBP, Systolic blood pressure; Scr, Serum Creatinine

†: Wilcoxon sign rank test was used to compare of differences within groups measured at baseline and 6 months

‡: Mann-Whitney U test was used to compare of differences between groups measured at baseline and 6 months

**Supplementary table 2 - Comparison changes in clinical parameters from baseline between different doses**

|  | **One pill (n=326)** |  |  |  | **Half a pill (n=142)** |  |  | **P-value**‡ |
| --- | --- | --- | --- | --- | --- | --- | --- | --- |
|  | **Baseline** | **6 months** | **P-value**† |  | **Baseline** | **6 months** | **P-value**† |  |
| HbA1c, % | 8.1 (7.5, 9.0) | 7.3 (6.9, 8.1) | <0.001* |  | 8.0 (7.5, 8.8) | 7.4 (6.9, 8.1) | <0.001* | 0.327 |
| Body weight, kg | 72 (64, 83) | 73 (62, 83) | <0.001* |  | 70 (62, 79) | 68 (60, 78) | <0.001* | 0.353 |
| SBP, mmHg | 139 (127, 151) | 134 (124, 146) | <0.001* |  | 142 (130,152) | 136 (125, 151) | 0.024* | 0.505 |
| DBP, mmHg | 78 (72, 89) | 78 (71, 87) | 0.013* |  | 79 (73, 87) | 78 (70, 85) | 0.125 | 0.843 |
| FPG, mg/dL | 167 (144, 194) | 138 (119, 158) | <0.001* |  | 158 (129, 184) | 134 (115, 157) | <0.001* | 0.221 |
| ALT, IU/L | 28 (21, 42) | 22 (18, 27) | <0.001* |  | 24 (18, 35) | 20 (17, 26) | 0.029* | 0.403 |
| eGFR, mL/ min/ 1.73 m^2^ | 83 (68, 98) | 81 (64, 96) | <0.001* |  | 83 (67, 98) | 81 (64, 98) | 0.395 | 0.120 |
| CHOL(T), mg/dL | 162 (140, 183) | 157 (138, 179) | 0.118 |  | 168 (145, 185) | 160 (138, 186) | 0.107 | 0.614 |
| Triglyceride, mg/ dL | 130 (91, 190) | 116 (80, 163) | <0.001* |  | 113 (82, 154) | 109 (76, 145) | 0.008* | 0.608 |
| LDL-C, mg/dL | 87 (72, 108) | 86 (70, 101) | 0.027* |  | 93 (77, 108) | 87 (74, 106) | 0.142 | 0.961 |
| HDL-C, mg/dL | 42 (35, 50) | 43 (36, 50) | 0.415 |  | 47 (39, 54) | 47 (38, 56) | 0.853 | 0.776 |

Data are shown as Median (IQR). ALT, alanine aminotransferase; CHOT(T), total cholesterol; DBP, diastolic blood pressure; eGFR, estimated glomerular filtration rate; FPG, fasting plasma glucose; HbA1c, Hemoglobin A1c; HDL-C, high-density lipoprotein- cholesterol level; LDL-C, low density lipoprotein- cholesterol level; SBP, Systolic blood pressure; Scr, Serum Creatinine

†: Wilcoxon sign rank test was used to compare of differences within groups measured at baseline and 6 months

‡: Mann-Whitney U test was used to compare of differences between groups measured at baseline and 6 months
